# Diversity, Abundance, and Ecological Roles of Planktonic Fungi in Marine Environments

**DOI:** 10.3390/jof8050491

**Published:** 2022-05-08

**Authors:** Kalyani Sen, Biswarup Sen, Guangyi Wang

**Affiliations:** 1 Center for Marine Environmental Ecology, School of Environmental Science and Engineering, Tianjin University, Tianjin 300072, China; ksen@tju.edu.cn (K.S.); bsen@tju.edu.cn (B.S.); 2 Key Laboratory of Systems Bioengineering (Ministry of Education), Tianjin University, Tianjin 300072, China; 3 Center for Biosafety Research and Strategy, Tianjin University, Tianjin 300072, China

**Keywords:** coastal, pelagic, water column, culturable fungi, metagenomics, biomass, mycoloop, biogeochemical cycling, nutrient metabolism

## Abstract

Fungi are considered terrestrial and oceans are a “fungal desert”. However, with the considerable progress made over past decades, fungi have emerged as morphologically, phylogenetically, and functionally diverse components of the marine water column. Although their communities are influenced by a plethora of environmental factors, the most influential include salinity, temperature, nutrients, and dissolved oxygen, suggesting that fungi respond to local environmental gradients. The biomass carbon of planktonic fungi exhibits spatiotemporal dynamics and can reach up to 1 μg CL^−1^ of seawater, rivaling bacteria on some occasions, which suggests their active and important role in the water column. In the nutrient-rich coastal water column, there is increasing evidence for their contribution to biogeochemical cycling and food web dynamics on account of their saprotrophic, parasitic, hyper-parasitic, and pathogenic attributes. Conversely, relatively little is known about their function in the open-ocean water column. Interestingly, methodological advances in sequencing and omics approach, the standardization of sequence data analysis tools, and integration of data through network analyses are enhancing our current understanding of the ecological roles of these multifarious and enigmatic members of the marine water column. This review summarizes the current knowledge of the diversity and abundance of planktonic fungi in the world’s oceans and provides an integrated and holistic view of their ecological roles.

## 1. Introduction

Fungi have long been known to be present in marine ecosystems [1], yet they are understudied compared to their terrestrial counterparts [2]. Over the last few decades, they have been formally accepted to form an ecological rather than a taxonomically defined group [3]. However, this overly restrictive ecological definition excludes the facultative marine fungi whose presence, growth, and survival in marine environments are well-established [4]. In addition, several deep-sea explorations have repeatedly brought to light the presence of a gamut of terrestrial fungi (Table 1) and also suggest their emergence and diversification in the ocean before that on land [5]. Therefore, the need for redefining marine fungi has been recently realized and emphasized. The development of a functional-scale classification by combining the existing definition of marine fungi with a three-level active and passive roles-based re-grouping was among the first to be suggested [6]. Thereafter, marine fungi have been defined either as those recovered repeatedly from marine habitats [7] or individuals with a long-term presence and metabolic activities in a marine habitat [8]. The former, currently the most complete definition, combines both genetic and functional aspects without relying on taxonomy [9]. Contrastingly, the later definition in an omics context seems user-friendly and simple and might be useful for revealing specific markers of fungal adaptation to marine environments. However, a consensus is yet to be reached on the definition of marine fungi.

The recent application of molecular approaches has revealed far more diverse and abundant marine fungi than those previously studied, with a growing body of evidence for their biogeochemical and ecological functions [18,19,20,21,22,23,24]. Furthermore, fungi isolated from marine or marine-related habitats are producers of several bioactive compounds [7,25,26,27,28,29,30], which can open up a new era of drug research. Nevertheless, marine fungi remain one of the most under-studied microbial groups, with 95% of the ocean remaining mycologically unexplored [9]. Consequently, the abundance, diversity, ecological roles, and interactions of marine fungi with other plankton remain mostly speculative and our current understanding of marine fungi, particularly planktonic fungi, remains diffuse. In this review, we provide a comprehensive summary of the diversity, abundance, and ecological roles of fungi in the marine water column, and highlight the knowledge gaps, and current and future trends in this topic. An integrated and holistic model illustrating the roles of fungi in the pelagic and benthic realms of the oceans is also presented.

## 2. Culturable and Molecular Diversity of Marine Fungi

### 2.1. Current Consensus of Culturable Diversity

Traditionally, marine fungi included higher (i.e., filamentous fungi in Basidiomycota and Ascomycota) and lower (i.e., zoosporic fungi in Chytridiomycota, Oomycetes, and Labyrinthulomycetes) fungi [31]. However, the latest update on their phylogeny has grouped them into evolved branches (Ascomycota, Basidiomycota, Blastocladiomycota, and Chytridiomycota) and basal lineages (Cryptomycota, Microsporidia, and Aphelida) [32]. The first inventory of cultured marine fungi described 209 species of higher filamentous fungi, 177 species of marine-occurring yeasts, and less than 100 species of the lower marine fungi [3]. This was followed by reports of 467 [33], 530 [34], 1112 [35], and 1257 [36] species of marine fungi. Currently, about 1900 marine fungal species, distributed across seven phyla (Aphelidiomycota, Ascomycota, Basidiomycota, Blastocladiomycota, Chytridiomycota, Mucoromycota, and Microsporidia), 22 classes, 88 orders, 226 families, and 769 genera, are documented (www.marinefungi.org, accessed on 1 May 2022). *Halosphaeriaceae* is the largest family of marine fungi, consisting of 141 species across 59 genera, and the most specious genera are *Candida* (64 species), *Aspergillus* (47 species), and *Penicillium* (39 species) [35]. The documented number (ca.1900 species) is much less than the estimated 10,000 species [33], which suggests that the oceans harbor a high fungal diversity, which is yet to be fully described.

### 2.2. Mycoplankton Diversity

#### 2.2.1. Microscopic Forms and Culturable Diversity

Fungi in the water column, commonly referred to as mycoplankton or planktonic fungi, were microscopically detected as individual filaments or hyphal aggregates, yeast forms, as well as picoeukaryote-associated and phytoplankton-associated zoosporic and cryptomycota forms [17,37,38,39,40]. The size range of individual filamentous forms is generally 1–3 μm in diameter and 10–200 μm in length [17,38], but in aggregate, they could reach up to 20 μm in diameter and >50 μm in length in coastal regions. The zoosporic forms (chytrids) in the coastal waters show a typical spherical sporangium (1–10 μm diameter) and rhizoid structure over 2 μm in length [41,42]. Some of these fungal forms with different lifestyles have been found to co-exist in the coastal water column [17]. The most common form of planktonic fungi encountered is yeast forms (size < 5 μm diameter), which have been found in a wide range of oceanic regions [43,44,45,46]. On the other hand, filamentous forms have been discovered mostly in coastal and coastal-upwelling regions [17,38,46].

Using culture-based methods, researchers characterized the culturable diversity of marine fungi mostly in nutrient-rich sediments. Those studies provided evidence for the presence of fungi in sediments, including subsurface, deep-sea, and anoxic sediments of different oceanic regions (Table S1). Apart from the most common ascomycetous and basidiomycetous fungi, several novel culturable fungi were also reported from marine sediments (Figure S1). Nevertheless, a vast majority of the fungi sampled from sediments are close to, or within, clades of terrestrial fungi (Table 1).

Most earlier studies revealed that a large proportion of culturable diversity in the water column comprised of yeasts, including *Rhodotorula*, *Rhodosporidium*, *Metchnikowia*, *Torulopsis*, *Kluyveromyces*, *Aureobasidium,* and *Cryptococcus* [43,44,45,46,47]. The common filamentous fungi and molds cultured from seawaters were *Aspergillus*, *Trichoderma*, *Arthrinium*, *Cladosporium, Penicillium*, *Cystobasidium*, *Exophiala*, *Graphium*, *Lecanicillium*, *Purpureocillium*, *Acremonium*, *Coniothyrium*, *Simplicillium, and Mucor* [46,48,49,50,51]. Yeasts and filamentous fungi were even reported from extreme habitats such as the hypersaline waters of Qatar, including the halo- and psychro-tolerant, red-pigmented yeast *Rhodotorula mucilaginosa*, and melanized filamentous fungi *Cladosporium* and *Alternaria* [52]. Filamentous fungi were also reported from the oil-spill-contaminated marine site where the predominant genera were found to be *Penicillium*, *Aspergillus*, and *Trichoderma* [53].

**Figure 1:**
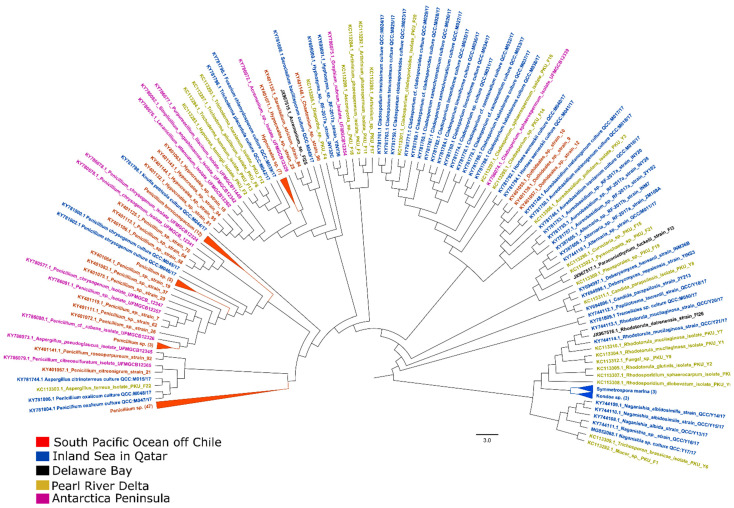
Maximum-likelihood (ML) tree of culturable fungi isolated from representative marine waters. A total of 192 ITS sequences of culturable fungi isolated from the water column (coastal and pelagic) across the globe were retrieved from the NCBI Nucleotide database. Sequences in the tree were aligned with MUSCLE using default settings. Phylogenetic analysis was performed using FastTree2.1 software (version 2.1, developed by Morgan N. Price, Berkeley, CA, USA) for the construction of the ML tree, which used the Shimodaira–Hasegawa test to estimate the reliability of each split in the tree. The sampling coordinates of the South Pacific Ocean off Chile [48], Inland Sea in Qatar [52], Delaware Bay [51], Pearl River Delta [49], and Antarctica Peninsula [50] are available in the corresponding publications.

This review provides a comparative analysis of the ITS (internal transcribed spacer) sequence diversity of the culturable fungi isolated from sediment and water samples of different geographical regions. Our analysis revealed that Ascomycota and Basidiomycota are the major phyla in both water (Figure 1) and sediment samples (Figure S1). The total sequence diversity of water samples was lower than that of the sediment samples, which could be a result of the poor availability of growth substrates in the water column or a low sampling effort. Interestingly, both filamentous and yeast forms of fungi were found in the global pool of culturable fungi isolated from the water column. The diversity of culturable fungal sequences determined in this study illustrates the consensus that marine fungi in the water column can range from yeast to filamentous forms.

Overall, culture-based studies indicate that mycoplankton diversity is limited to filamentous fungi and ascomycetous and basidiomycetous yeasts. The probable reasons for such a seemingly low diversity could be less sampling effort or nutrient-poor water column. Moreover, culture-based studies are known for their inherent biases, including the selective enrichment of a few phyla and difficulty in isolating host-associated fungi.

#### 2.2.2. Molecular Diversity and Dynamics of Mycoplankton

Past culture-based studies have revealed the presence of yeasts and filamentous fungi. However, they failed to discover the zoosporic fungi in the marine water column. On the contrary, high-throughput sequencing (HTS) efforts revealed a lot more diversity, including the prevalence of zoosporic fungi, in several marine habitats [18,19,21,42,46,54]. Moreover, molecular surveys of marine eukaryotes detected fungi not only in the euphotic zone of the global ocean [55] but also in the entire water column [56]. Unfortunately, molecular surveys of eukaryotes could not provide any evidence for the extent of fungal diversity in the coastal and open-ocean waters.

Over the last decade, efforts were made to investigate the diversity of mycoplankton in both coastal and open-ocean waters (Table 2). The spatial analyses of planktonic fungi, based on DNA fingerprinting, could reveal the dynamics of positive fungal genotypes [38,57] and the presence of only Dikarya [58]. Especially in Hawaiian coastal waters, the exclusive presence of Dothideomycetes (four species) and dominance of Basidiomycota, including several novel phylotypes (42 species), were documented. The fungal communities displayed a noticeable spatial (lateral and vertical) diversity, with the vertical diversity profile being different for coastal and open-ocean waters [58]. Similarly, in the upwelling ecosystem off the coast of Central Chile, the fungal diversity was distinct, with a higher richness at the near-shore site than that of the off-shore site and a tendency to decrease with depth [57]. However, due to the inherent biases of fingerprinting techniques, this could only provide a limited view of fungal diversity. With the application of HTS, recent studies provide a deeper assessment of planktonic fungal communities and uncover many OTUs, classified into a wide range of phyla and several unclassified and possibly novel fungi from coastal waters (Table 2). Most of these studies documented the predominance of Dikarya and the prevalence of Chytridiomycota in coastal waters. However, a few studies also provided evidence for the occurrence of Cryptomycota (also known as Rozellomycota), Mucoromycota, Glomeromycota, and Neocallimastigomycota. Overall, the HTS approach provided evidence for the presence of zoosporic and basal phyla and altered the earlier notion that Dikarya fungi are exclusive inhabitants of the ocean.

Apart from the spatial variations of mycoplankton discussed above, some studies described the temporal dynamics of fungi in the coastal water column. For example, a multi-year assessment study of coastal waters at Plymouth found that Dikarya and Chytridiomycota were both dominant and dynamic, with several abundant and dominant orders [19]. Similarly, another multi-year study of fungal diversity at Piver’s Island Coastal Observatory (PICO), USA, a coastal mesotrophic ocean site, showed not only the dominance of Ascomycota but also interannually indicated seasonal patterns of Basidiomycota, Chytridiomycota, and Mucoromycotina [21]. Particularly, Chytridiomycota (order Rhizophydiales) and Mucoromycotina were detected in winter and Glomeromycota in early winter and spring. In addition, the highest richness and diversity of fungi during winter and the lowest during summer were detected at PICO. Contrastingly, in the coastal waters of the Bohai Sea, Chytridiomycota (order Rhizophydiales) dominated Ascomycota and Basidiomycota in April, indicating a possible association with phytoplankton bloom [18]. Temporal changes in the community composition of fungi were also evident during different stages of algal bloom in the coastal waters of Shenzhen [59]. Several genera prevailed in the pre-bloom stage; however, only *Malassezia* dominated the onset and the peak bloom stages. *Saitoella* and *Lipomyces* gradually succeeded *Malassezia* and eventually, *Rozella* dominated the terminal stage. Notably, the bloom decline stage exhibited a higher diversity than the pre-and peak-bloom stages. Collectively, the above time series studies suggest that fungi respond to seasonality and phytoplankton dynamics, which supports the view that they are residents of the coastal water column and are most likely metabolically active biomass.

Similar to the coastal water column, several lines of evidence indicated a high molecular diversity of fungi, including several unidentified and potentially novel species, in the open-ocean water column. For example, a high diversity of fungi, with the predominance of Dikarya, was reported for the first time in waters of the open-ocean transect from the Hawaiian coast to Australia [64]. Within Ascomycota and Basidiomycota, the family Nectriaceae and genus *Malassezia,* respectively, were the most common open-ocean fungi. Unfortunately, only Dikarya were documented, probably due to the insufficient coverage of the clone libraries. However, later studies that adopted HTS additionally uncovered several basal phyla (Table 2). For example, a study of the epi- to abyssopelagic zone of the Western Pacific Ocean documented OTUs that were assigned to Ascomycota, Basidiomycota, Chytridiomycota, and Mucoromycota, with Ascomycota as the most dominant phylum [23]. Furthermore, the classes Sordariomycetes, Eurotiomycetes, Dothideomycetes, Saccharomycetes, and the order Malasseziales were found to dominate the fungal communities. Compared to other zones, a higher OTU richness and distinct fungal community were evident in the epipelagic zone. Yet, another study of the water column suggested an increasing number of OTUs of the ascomycetous genus *Aspergillus* from coastal to open-ocean waters [54]. Contrastingly, in the waters of the South Pacific Ocean, Chytridiomycota (order Rhizophydiales) was reported as one of the dominant fungi. The occurrence of chytrids in oceanic waters suggested that their ecological importance in open oceans was similar to that in coastal water columns [61].

In summary, most molecular surveys of planktonic fungi report the dominance of Dikarya and suggest that many fungal OTUs in both coastal and open-ocean waters are yet to be described. Furthermore, by reprocessing more than 600 HTS datasets and analyzing 4.9 × 10^9^ sequences (4.8 × 10^9^ shotgun metagenomic reads and 1.0 × 10^8^ amplicon sequences), a recent study found that every fungal phylum is represented in the global marine planktonic mycobiome [65]. However, the global marine mycobiome is generally predominated by Ascomycota, Basidiomycota, and Chytridiomycota. Particularly, the coastal and open-ocean fungal communities show the dominance of ascomycetous classes, such as Sordariomycetes, Eurotiomycetes, Dothideomycetes, Saccharomycetes, and Pezizomycetes. These findings corroborate previous culture-based studies, which report the prevalence of members of classes Dothideomycetes and Sordariomycetes in mangroves and coastal waters [66,67]. These classes of fungi are suggested to have adaptations (dispersal and attachment) for sustenance in marine environments [36,68]. Contrary to ascomycetous fungi, basidiomycetous fungi appear scarce, with Ustilaginomycetes, Agaricomycetes, Exobasidiomycetes, Wallemiomycetes, and Tremellomycetes being generally detected [18,19,20]. Interestingly, molecular surveys uncover a richer diversity of basidiomycetous classes than culture-based methods, where only Exobasidiomycetes, Agaricomycetes, and Ustilaginomycetes are described [36]. Furthermore, only *Pleosporales, Dothideales, Capnodiales, Eurotiales, Malasseziales*, *Hypocreales,* and *Rhizophydiales* appear ubiquitous from molecular surveys, despite the 74 known orders of culturable marine fungi [36]. The diverse and dynamic patterns of fungi in oceanic waters similar to nutrient-rich coastal waters, which emerged from molecular surveys, raise questions about their modes of nutrition and roles in oligotrophic conditions. More importantly, the differences in the abundances evident across space and time support the proposition that planktonic fungi are viable and responsive to environmental changes.

### 2.3. Environmental Drivers of Mycoplankton Diversity

Environmental factors are known to play an important role in regulating microbial community structure and diversity [69,70]. In terrestrial realms, fungi have unique requirements, and species segregate along environmental gradients [71,72]. Likewise, several lines of evidence suggest the role of environmental factors in shaping the fungal diversity of the water column (Table 3). For example, phytoplankton and primary production, nutrients, salinity, organic matter, seasonality, DO, and temperature have been reported as the key factors that govern mycoplankton diversity. In parallel, it has been suggested that riverine inputs of fungi might be responsible for a higher fungal richness in coastal sites than that in off-shore sites [57]. The other less-reported environmental factors such as ocean currents, hydrographic conditions, depth, DO, COD, nitrate, flow, conductivity, insolation, pH, DIC, oxygen concentration, riverine inputs, tidal actions, dispersal, and biological interactions were also shown to influence fungal communities of seawater columns [17,20,21,54,59,61,73,74]. These environmental associations of mycoplankton can potentially have several ecological implications, including spatiotemporal variations, organic matter decomposition, niche differentiation, host–parasite interactions, and the regulation of phytoplankton bloom (Table 3), which are yet to be fully established. Undoubtedly, the associations of fungi with a multitude of environmental factors, evident from the above studies, suggest that fungi respond to environmental gradients, and their communities can be shaped by local conditions. Although significant differences among oceanographic regions were identified, latitudinal gradients of the richness and diversity of marine fungi were not observed [65]. This was unlike the pattern observed for planktonic marine bacteria [75]. Perhaps with the availability of more HTS datasets, it would be essential to expand the collection of reference loci and genomes to determine the typical environmental drivers of planktonic fungi [65].

## 3. Abundance of Mycoplankton

A typical milliliter of seawater is known to contain about 1000 fungal cells [77]. The abundance of fungi has been often estimated by researchers using culturable, microscopic, or molecular methods. However, due to ‘great plate anomaly’ and other biases, the densities of culturable fungi in the ocean are several orders of magnitude lower than that of fungi detected either by direct detection or molecular techniques. The culturable fungal abundance (CFU L^−1^) was found to be three orders of magnitude [78,79], while the abundance (gene copies L^−1^) based on the qPCR method was five to eight orders of magnitude [18,19,80,81]. Fungal enumeration by culturing has been criticized because a colony can arise out of single spores, groups of spores, single cells, or mycelial fragments. Therefore, methods based on direct detection of fungal hyphae or ergosterol and qPCR have been developed (Table 4). Even though these alternative methods have their own biases, they are much less time-consuming and labor-intensive and provide reasonably reliable estimates of fungal abundance.

Fungal filaments, ranging from 1–3 μm in diameter and 10–200 μm in length were detected as individual filaments or aggregates in the coastal upwelling ecosystem off the coast of Central Chile using the Calcofluor White staining method. This aggregate formation was associated with the efficient remineralization of organic matter in seawater [38]. The vertical profile of fungal biomass showed higher values at the surface compared to greater depths and agreed with those of phytoplankton biomass and physicochemical parameters, suggesting higher fungal activity during high organic matter availability in a coastal upwelling ecosystem off the coast of Chile [82]. In the same study, the fungal biomass determined by the abundance of hyphae positively correlated with phospholipid fatty acid (18:2ω6), a fungal biomarker, and reflected the degradation of protein and carbohydrate polymers. Of interest, the fungal biomass (0.04 μgCL^−1^ to 40 μgCL^−1^) was comparable to prokaryotic biomass (10 μgCL^−1^ to 44 μgCL^−1^) and both biomasses peaked upon a decline in phytoplankton biomass, suggesting that the availability of detritus determined their abundances. Such an association of fungal abundance was also evident from studies that were based on molecular techniques [19,21].

The analysis of the abundances of major planktonic fungi (Ascomycota and Basidiomycota) in a transect from the Hawaiian coast to Australia revealed that Ascomycota had a high abundance only in coastal stations, whereas Basidiomycota was high in both oceanic and coastal stations [64]. The abundance of Basidiomycota (maximum 10 ng/μL, open-ocean station) was much higher than that of Ascomycota (maximum 14 pg/μL, coastal station) and similar to that of bacterioplankton in all the stations. The abundance of mycoplankton was highest at the surface, a pattern similar to that exhibited by bacterioplankton in most stations. In a high-resolution time-series study at PICO, an abundance of up to eight orders of magnitude was observed with two peaks each year, one each in summer and fall. The abundance was found to exhibit a dynamic pattern and was linked to chlorophyll *a*, SiO4, and oxygen saturation. As PICO is a site with a high salinity, no correlation was observed between abundance and salinity [21]. Conversely, mycoplankton abundance was shown to positively correlate with particulate organic carbon, ammonia, total particulate nitrogen, and particulate organic nitrogen, while negatively with salinity at the coastal Plymouth site [19]. The negative correlation with salinity was attributed to an increased abundance due to riverine inputs. Whereas the factors with a positive correlation were the growth substrates that increase with autochthonous production or allochthonous inputs [19]. Sites that experience river inflows are generally reported to contain fungal and nutritional inputs from terrestrial sources [38,51,85].

Taken together, these studies reveal the ubiquitous presence and high abundance of mycoplankton within marine environments. Evidence of mycoplankton abundance similar to that of bacterioplankton in nutrient-rich habitats emphasizes that mycoplankton are an important component of coastal realms. The association of mycoplankton with environmental factors suggests their important role in detrital processing and nutrient cycling. The paucity of knowledge on mycoplankton abundance patterns in the pelagic realm warrants future investigations.

## 4. Ecological Roles of Mycoplankton

**Figure 2:**
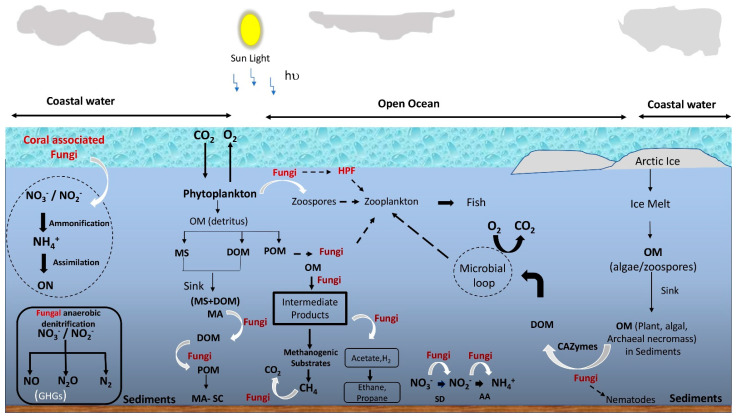
Schematic representation of the overview of possible roles of fungi in the marine food web and biogeochemical cycling. OM = organic matter; DOM = dissolved organic matter; MS = marine snow; POM = particulate organic matter; HPF = hyper parasitic fungi; SD = suboxic denitrification; AA = anaerobic ammonification; GHG = greenhouse gas; MA = macroaggregate; MA-SC = macroaggregate-sequestered carbon; ON = organic nitrogen. Black dotted arrows indicate feeding and white curved arrows indicate fungal involvement in the conversion.

Fungi in the transition zones of salt marshes and mangroves were found to play the roles of saprobes, symbionts, pathogens, and parasites, similar to their terrestrial counterparts [36]. Currently, their roles are yet to be established, especially in the open-ocean water column, even though they have been detected in the entire marine water column. Arguably, a reliance on osmotrophy determines the ecology of fungi in marine ecosystems similar to terrestrial, and therefore nutrient-rich environments have both abundant and diverse fungi. In addition, the ability to attach to detritus or particulate organic matter enables fungi to grow in the flowing and turbulent water of the oceans. Thus, owing to these two important aspects of fungal feeding strategies (i.e., osmotrophy and attachment to the substrate), marine fungi are generally considered to play the roles of decomposers, parasites, and denitrifiers [12,86]. Unfortunately, only a few studies provide direct evidence of their ecological roles; thus, fungi are often neglected in the ocean ecosystem models [64,87]. Nevertheless, with the piling evidence of their contribution to the marine ecosystems, marine microbiologists have started to realize their importance in nutrient cycling and the food web. For example, laboratory-based physiological studies [88], biomass [89], direct detection of fungal mycelia [10,82], zoospores and rhizoid structures on host cells [41,42], metabolic potential/physiological diversity analysis [90], the high copy number of rRNA gene [19,64,81], live fungal biomass (ergosterol) [20,24] provide indications of their viability and possible ecological roles in the water column. The following sub-sections discuss the predicted ecological roles of fungi in marine environments, which are illustrated in Figure 2.

### 4.1. Biogeochemical Cycling

#### 4.1.1. Role in Organic Matter Decomposition and Aggregation

Marine ecosystems receive and process a large amount of bio-recalcitrant, terrigenous organic matter (particulate) often in the form of lignocellulosic substrates. In addition, a large pool of bio-labile organic matter (dissolved and particulate) in the ocean is produced from algal detritus. These forms of organic matter in the ocean are mainly recycled by microbial decomposers such as bacteria and fungi. Compared to bacteria, fungi can more efficiently mineralize lignocellulosic substrates due to their lower metabolic nutrient demand and wider enzymatic capabilities [91]. The decomposer role of fungi in aquatic ecosystems is mainly known from lotic systems, mangroves, and wetlands [92,93,94]. However, the frequent isolation of marine fungi from floating, sunken woody substrates, and plant detritus [95,96] also suggests such a role in coastal and pelagic ecosystems.

The colonization of lignocellulosic substrates by marine fungi is extensively studied [97]. However, there are fewer studies on their ability to utilize the lignocellulosic materials in the environment. In vitro studies suggest that marine fungi have the potential to degrade lignocellulosic components by their ability to produce hydrolytic enzymes, such as laccase, cellulase, amylase, alginase, laminarinase, peroxidase, pectinase, and xylanase [98,99,100,101,102]. Marine ascomycetes and basidiomycetes were demonstrated to solubilize significant amounts of lignin from wood in vitro, suggesting that they can carry out a ‘white-rot like’ role in the marine environment [103,104,105,106]. For example, the basidiomycetes *Nia vibrissa*, which were isolated from wood submerged in the sea, caused a pattern of wood decay characteristic of the white-rot type when cultured on different wood species [104]. Historically, the morphological decay features observed in woody biomass colonized by marine fungi were indicative of soft-rot and white-rot decay [107]. The soft-rot strategy is efficient in marine systems compared to white rot, which suffers from the leaching of lignocellulolytic enzymes into the surroundings [97,100,108,109]. Typically, the soft-rot strategy involves extensive cellulose and hemicellulose degradation with limited lignin degradation, and such a strategy is key to the survival of ascomycetes in oceanic waters [109]. The prevalence of the soft-rot strategy in oceanic waters was evident from the dominance of ascomycetes and disappearance of basidiomycetes with the prolonged submersion of the woody substrate (driftwood) in the Arctic Ocean [109]. The above findings perhaps advocate that these marine fungi, armored with lignocellulolytic activity, are most likely capable of degrading lignocellulosic substrates in both coastal and oceanic waters by colonization.

Apart from lignocellulosic substrates, marine fungi are also capable of processing algal polymeric substrates by secreting a plethora of hydrolytic enzymes in vitro [101]. It has been shown that the hydrolytic activity of fungi increases in presence of phytoplankton-derived biopolymers, and such activity can process about 30% photosynthetic carbon in a coastal upwelling system [82]. Moreover, in the coastal water column, fungi are found to grow during productive periods of high substrate availability and feature high hydrolytic activity [82]. Later experiments demonstrated the assimilation of ^13^C-labeled algal transparent exopolysaccharides (TEP) and the accumulation of ^13^C in *Cladosporium* (Ascomycota) and *Malassezia* (Basidiomycota), which provide direct evidence for the utilization of algal polysaccharides by saprotrophic planktonic fungi [110].

Some studies show that fungi in marine environments produce macroaggregates from DOM without the need for nucleation, where the presence of fungal hyphae makes the macroaggregates stable and renders them less easily degradable [89]. Such evidence of macroaggregates in deep-sea regions is predicted to lead to long-term carbon sequestration, ultimately affecting the carbon biogeochemical cycling and global weather change [89]. A similar aggregate formation was also observed in the coastal water column, and it was suggested that the combined action of fungi and bacteria could result in a highly efficient microbioreactor able to process particulate organic matter (POM) and DOM during sedimentation [38]. Furthermore, it is speculated that fungi contribute to organic matter degradation in the deep sea owing to their dominance in the overall biomass within marine aggregates (snow) [111]. These findings suggest that planktonic fungi play a role in the formation and stabilization of the marine aggregates and their simultaneous degradation to DOM. Interestingly, such a contribution highlights their possible link to the POM-DOM cycling in the ocean (Figure 2). Thus, fungi might play a much more important role in biological carbon pumps or ocean carbon storage than what is currently perceived.

As fungi are known to produce a variety of enzymes that have the potential to break down the chemical bonds of plastic polymers, they might have a role in the degradation of marine plastics [112]. Seminal works on plastic deterioration by marine fungi have suggested that polyurethanes are more susceptible to fungal attacks [113]. Interestingly, a recent study reported that fungi (e.g., *Aspergillus flavus*, *A*. *terreus*, *A. niger*, *A. fumigatus*, and *Penicillium* sp.) isolated from seawater are potential degraders of polyethylene [114]. These reports highlight the underestimated role of planktonic fungi as degraders of marine plastic wastes.

Considering earlier studies, it is not surprising that marine fungi can carry out the role of saprotrophs in the coastal and ocean waters. With their unique ability to produce a myriad of hydrolytic enzymes and the colonization of lignocellulosic substrates, algal biopolymers, marine snow, and plastics, marine fungi might contribute to the process of microbial carbon sequestration in the ocean. Their role in long-term carbon sequestration, however, remains speculative and needs further investigation.

#### 4.1.2. Role in Nutrient Metabolism

With the recent application of omics and microarray techniques, our ability to understand the mechanisms underpinning the function of marine fungi in biogeochemical cycling is accelerating. Particularly, studies based on metagenomics and metatranscriptomics provided evidence for the fungi-associated metabolic processes in the marine and water columns. Metagenomic studies discovered genes involved in amino acid metabolism, the aerobic carboxylation of glucose, anaerobic decarboxylation of pyruvate, urea, sulfur metabolism, etc. [6,22]. Fungal genes involved in complex C and fatty acid metabolism have been found across all depths and regions, and it is suggested that fungi might replace phytoplankton for vitamin supplies in deep waters [22]. Similarly, metatranscriptomics also revealed fungal transcripts that were assigned to protein, carbohydrate, and lipid metabolism [115]. Some studies based on metatranscriptomics prove the presence of only fungal carbohydrate-active enzymes (CAZymes) and carbohydrate-binding modules in the secreted proteome, suggesting active carbohydrate (microbial cell envelopes, plant, and algal detritus) degradation by fungi and their involvement in carbon cycling in the ecosystems [116]. Metagenome prediction using the PICRUSt2 tool suggests that fungal communities in marine waters are primarily aerobic and acquire energy through the oxidation of fatty acids [117]. This study also suggested that the metabolism of amino acids, carbohydrates and energy, fatty acids and lipids, nitrogen, sulfur, and other compounds, such as vitamins, octane, methyl ketone, heme, and secondary metabolite, possibly represent the core metabolism of marine mycoplankton in marine habitats ranging from estuarine to open ocean. In addition, high CAZymes per gene suggested that pelagic fungi are active in carbohydrate degradation [118].

A few earlier studies have shown the presence of fungi of known and new taxonomic groups in methane hydrates [119], suggesting their possible role in carbon flux fueled by methane, similar to the methanotrophic prokaryotes [120]. Although, methane-utilizing yeasts were reported much earlier [121], a more recent study revealed the significant correlation of the members of marine yeast, *Cryptococcus curvatus*, with methane and ethane [122]. This suggests the involvement of fungi in methane cycling in the ocean and their probable interactions with methanogenic or ethanogenic prokaryotes. In addition, fungi are proposed to be H_2_ producers that help in the growth and survival of sulfate-reducing bacteria in the deep ocean, indicating their possible involvement in the anaerobic oxidation of methane [123]. A few studies also suggested the role of fungi in nitrogen cycling in the ocean. Marine fungi were found to be associated with nitrate reduction, nitrite accumulation, and ammonia formation in the anoxic region of the ocean [88], denitrification, co-denitrification, ammonification [124], and nitrite reduction in the deep biosphere [115]. Using GeoChip, several fungal genes were detected that catalyze ammonification from nitrite and urea, ammonia assimilation, and denitrification in marine sediment [24]. Another line of evidence suggests that endolithic fungi were involved in at least two processes of the nitrogen cycle within corals: (1) reduction of nitrate and/or nitrite to ammonia, and (2) ammonia assimilation for biosynthesis [125]. As marine fungi are capable of anaerobic denitrification with the formation of greenhouse gases (NO and N_2_O) and nitrogen (N_2_) [126], their impact on global climate should be further explored.

Overall, these findings suggest fungi as an important component of nutrient cycling (both carbon and nitrogen) in the ocean and warrant their inclusion in marine microbial ecosystem models involving biogeochemical cycling.

### 4.2. Fungal Contribution to the Marine Food Web and Biotic Interactions

Mycoplankton are known to play an important role within the marine microbial food web as diatom parasites [17,19,41]. Particularly, chytrids found in the coastal water column, open ocean, and Arctic regions are reported to channel organic matter and energy to higher trophic levels converting inedible phytoplankton to zoospores (high in polyunsaturated fatty acids and cholesterol) that serve as food for zooplankton [42,127,128]. This mechanism, known as the mycoloop, provides nutrients to the food web through the zoospores of either parasitic fungi or saprotrophic fungi. Fungi feed on substrates inedible for zooplankton, and in turn, produce zoospores rich in nutrients that are palatable to zooplankton [94]. These zoospores become a major food source, especially when inedible food sources predominate, thus making fungi responsible for the growth and reproduction of zooplankton [110,129]. The non-grazed zoospores, in turn, might contribute to the DOM and the detritus pool [128]. In addition, the fungi that are diatom parasites might prove to be successful competitors against zooplankton by controlling energy flow and food web dynamics [130]. Thus, the fungus–zooplankton association may alter the food web dynamics by either increasing the population of zooplankton or decreasing it. Furthermore, fungi may also serve as hosts for hyper-parasites, thereby reducing the parasitic load on the phytoplankton, and owing to their smaller size, hyperparasites, in turn, are grazed by zooplankton [131]. A tripartite interaction between Cryptomycota (hyperparasite), Chytridiomycota (parasite, saprotroph), and phytoplankton [18], and the niche separation between Cryptomycota (algal parasite) and Chytridiomycota have been speculated [132]. A recent study also revealed that Rozellomycota fungi, which are dominant during pre- and early bloom stages, have the potential to fuel a marine mycoloop [133]. Direct evidence of fungal parasitism in the marine water column, especially in the oceanic water column, is scarce, and thus would be an interesting topic of further exploration.

The organic detritus and its associated microbes are important to the marine food web. Fungi can convert the detritus into palatable forms for detritivores owing to their lignocellulose degradation capability, and are thus suggested to play an important role in the coastal water column and/or open-ocean detrital dynamics [82,95]. Fungi and bacteria occupy different functional niches in the decomposition of POM, wherein fungi act as primary degraders of particulate, and bacteria act as rapid recyclers of nutrient-rich organic matter compounds (e.g., algal biopolymers) [93]. Towards the decaying stages of a diatomic bloom, diatom secretes a large amount of mucus that forms aggregates (marine snow) in the water column, which are likely to act as chemical cues for colonization by fungal zoospores. These aggregates contribute to pelagic detritus, and upon sedimentation, they are transported along with the attached fungi to the deep sea [82]. Similarly, the colonization of transparent, exopolymeric particles (TEPs) by fungal hyphae was also observed, suggesting the possible transportation of the fungal mycelia-bound TEPs to the ocean’s sediment [10]. Marine snow or TEP-associated fungi possibly re-mineralize the polysaccharides therein and contribute to the bulk of DOM in the deep sea [95]. As marine snow aggregates are recalcitrant to bacterial degradation, the saprotrophic action of fungi supports bacterial metabolism by making DOM available to bacteria. Another piece of evidence for the fungal utilization of algal TEPs suggests the possible interactions (e.g., competition and syntropy) between bacteria and planktonic fungi [110,134]. Although fungi and bacteria were found to co-exist in the water column and serve as food sources for zooplankton, the role of fungi might be more significant than bacteria as they prevent decoupling between primary and secondary production and transfer carbon up the marine food web [135].

Marine fungi were acknowledged for their great importance under ocean acidification [79]. In fact, under ocean acidification, a reduction in Chytridiomycetes and Cryptomycota was reported [136]. It was further suggested that a decrease in the number of these parasitic members might lead to a subsequent increase in large phytoplankton and a decrease in small phytoplankton. This would alter the food web structure and may lead to a decrease in zooplankton. Moreover, an increase in the abundance of pathogenic fungi was also observed with acidification [136]. Several lines of evidence gathered from culturable and molecular studies of water columns suggest the presence of fungi that are known pathogens of plants, vertebrates, and invertebrates [23,50,59,136]. Thus, there might be an increase in pathogenic fungal abundance with progress in ocean acidification, leading to the breakdown of ecosystems. Planktonic fungi are also susceptible to viral infection, and because viruses are abundant (10^7^–10^8^ particles/mL) in marine environments, their lysis of planktonic fungi might also contribute to food web dynamics [137]. A few studies report the presence of mycoviruses in marine ecosystems and that the viral lysis of fungi might contribute to another pathway of carbon flow into the DOM pool [24,138]. However, further investigations are needed to clearly understand the interactions between fungi and viruses in the water column.

Marine fungi are the components of a complex matrix of multipartite interactions and play an important role in the food web as both saprotroph (consumer) and progenitor of zoospores (secondary producer). Further studies involving empirical dynamic modeling approaches, such as linear (multiple autoregressive models) and non-linear (convergent cross-mapping) models, can shed light on food web dynamics by generating data for a network analysis of such chaotic systems [137]. Currently, network analyses have mainly been used to understand the spatial distribution of marine fungal OTUs among sampling sites [12], sea regions and temperatures [139], and competitive and cooperative relationships within OTUs [18]. Additionally, network models could decipher the relationships within fungal taxa, and between fungi and other eukaryotes (primary producers, fungal predators) cohabiting a freshwater lake [140]. Nevertheless, the modeling approaches mentioned above can also contribute to the understanding of the host–parasite relationship and its consequences on the food web, estimation of energy and matter transfer in the food web, and co-occurrence of fungal groups and their correlation with physicochemical and biological variables [12,128,137,139].

## 5. Future Perspectives

This review summarizes our current knowledge of planktonic fungi and provides valuable insights into their ecological importance in coastal and pelagic realms. Currently, the research on diversity, abundance, and the role of planktonic fungi is still in its infancy and limited to very specific geographical regions. So far, studies are confined mostly to the coastal water column and only a few have addressed the open-ocean water column. To this end, global explorations, especially for open-ocean waters, are needed to shed new light on the ubiquitous (generalist) and localized (specialist) planktonic fungi. From a methodological point of view, the study of fungi in the ocean is witnessing a paradigm shift from culture-based to molecular surveys with the ITS region as the fungal barcode. However, the choice of fungal markers is known to limit the extent of diversity in molecular surveys, suggesting that primer specificity, coverage, and bias should be addressed before their application. Furthermore, DNA surveys have been criticized for their overestimation of diversity due to the inclusion of relic DNA. Conversely, the RNA surveys that provide information on the active fungi are seemingly more appropriate. Considering the above biases, a polyphasic approach is expected to provide an accurate estimate and comprehensive view of planktonic fungal diversity.

Planktonic fungi are presumed to play a significant role in organic matter transformation, prevent the decoupling of primary and secondary production, and transfer carbon to higher trophic levels. Particularly, the discovery of fungal parasitism and mycoloop amidst issues such as polar ice melting, global warming, and ocean acidification possibly indicates the potential of fungi to restructure the marine food web by modulating carbon flow. This further suggests the importance of the effect of fungal parasitism and warrants its inclusion in ocean ecosystem models. To this end, studies that focus on (1) molecular detection and the enumeration of both parasitic and hyper-parasitic fungi, (2) life-table experiments to study trophic flow, and (3) carbon transfer among phytoplankton, saprotrophic and parasitic fungi can provide a deeper understanding of fungal parasitism and its effects on the marine food web.

Currently, the questions that remain to be fully answered are: (1) what is the extent of planktonic fungal diversity? (2) What roles do undescribed fungi play in the coastal and oceanic waters? (3) Are there any keystone species in the planktonic fungal community? (4) How do planktonic fungi interact with other components of the food web? (5) How much carbon do they recycle, and (6) how would they affect the global climate and vice versa? Future studies involving genomics, metagenomics, transcriptomics, and metatranscriptomics would help to answer these key questions. Interestingly, the discussions in recent reviews on the adaptation of marine fungi in the marine environment might help to reduce the impacts of climate change on marine organisms and environments [141,142]. With the inflow of new information, the haze would clear around planktonic fungi, providing insights into the black box of their cryptic presence, role, and significance in the marine water column. This review is expected to provide a holistic view of planktonic fungal ecology and a framework for future research in this area.

## Figures and Tables

**Table 1:** List of fungi of terrestrial origin discovered through culture-based and molecular methods from different oceanic regions.

Terrestrial Fungi	Method	Sampling Region	References
*Aspergillus, Penicillium, Cladosporium, Fusarium, Sagenomella, Exophiala, Tilletiopsis,*	Culture-based	Central India basin	[10,11]
*Fusarium, Aspergillus, Phoma, Cladosporium, Mortierella, Sebacina, Alternaria*	454 pyrosequencing	Kongsfjorden (Svalbard, High Arctic)	[12]
*Fusarium, Acremonium, Penicillium, Aspergillus Cladosporium Rhodotorulla, Paecilomyces, Exophiala, Meyerozyma*	Culture-based	Canterbury Basin sediments, New Zealand	[13]
*Malassezia*	RNA- based clone library	Peru	[14]
*Thelephoraceae, Trichophaea*	Illumina MiSeq sequencing	Southwest India Ridge	[15]
Mycorrhizal fungi (*Ambispora, Claroideoglomus, Diversispora, Glomus, Funneliformis*)	Illumina HiSeq sequencing	East China Sea	[16]
*Malassezia, Nectria, Acremonium, Leptosphaeria, Candida and Clavispora*	ITS-clone library	Hawaiian waters	[17]
Mortierellales	Illumina HiSeq sequencing	Bohai Sea water column	[18]

**Table 2:** Application of high-throughput sequencing (HTS) methods in the assessment of fungal diversity of marine water columns.

Method	Target Region	Primers	Number of OTUs	Phyla	Sampling Region	Reference
454 Pyrosequencing	18S (V4)	TAReuk454FWD1 and TAReukREV3	71	Chytridiomycota and Dikarya*	European near-shore sites	[40]
454 Pyrosequencing	18S (V4)	TAReuk454FWD1 and TAReukREV3	23,263 seqs.	Chytridiomycota, Dikarya, and Cryptomycota	Arctic and temperate biomes	[60]
454 Pyrosequencing	ITS	ITS1F and ITS4	-	Coastal water: Chytrids (36%) Open ocean: Rhizophydiales (30%)	Tasman Sea, and East Australian Current	[61]
454 Pyrosequencing	ITS1	ITS1F and ITS2	3468	Dikarya, Chytridiomycota, Mucroromycotina, and Cryptomycota	Dongchong Bay, China	[59]
Illumina HiSeq	ITS1	ITS1F and ITS2	1483	Dikarya, Chytridiomycota, Mucoromycota, and Cryptomycota	Bohai Sea	[18]
Illumina Hiseq	ITS	528F and 706R	91	Dikarya, Glomeromycota, Chytridiomycota, and Cryptomycota	Mariana Trench	[62]
Illumina Hiseq	ITS2	ITS3 and ITS4	8701	Dikarya, Chytridiomycota, Glomeromycota, and Rozellomycota	East China Sea water column and sediments	[54]
Illumina Hiseq	ITS2:	ITS3 and ITS4	4028	Dikarya, Chytridiomycota, and Mucoromycota	Western Pacific Ocean (Epi-Abyssopelagic zone)	[23]
Illumina MiSeq	ITS	ITS1F and ITS4	582	Dikarya and Chytridiomycota	Plymouth, UK	[19]
Illumina Miseq	ITS	ITS1F and ITS4	2796	Dikarya and Chytridiomycota, Glomeromycota, and Neocallimastigomycota	Piver’s Island Coastal Observatory (PICO), USA	[21]
Ion-Torrent	LSU	LR0R and EDF360R	2305	Ascomycota, Basidiomycota, and Chytridiomycota	Piver’s Island	[63]

* Dikarya: Ascomycota and Basidiomycota.

**Table 3:** Factors affecting fungal assemblages in water columns of different marine habitats and their ecological implication.

Strongly Correlated Factors	Region	Ecological Implication	Reference
Chlorophyll *a*, temperature, phytoplankton biomass	Hawaiian coast	Spatial variations	[58]
Phytoplankton, nutrients (nitrate, phosphate, nitrite), and location	West Pacific Warm Pool	Organic matter decomposition	[64]
Chlorophyll *a*, organic matter, and warm conditions	Upwelling ecosystem off the coast of Central Chile	Organic matter decomposition	[38]
High nitrogen availability, reduced salinity, temperature, phytoplankton, organic matter	Coastal station off Plymouth	Temporal variations, niche differentiation, and host–parasite interactions	[19]
Salinity, temperature, oxygen, and nutrients	Tasman Sea, East Tasman Sea, and East Australian Current	Biogeochemical cycling and spatial variations	[61]
Depth, dissolved oxygen, and nitrate	Across the globe	Local environmental conditions govern assemblages	[73]
Temperature, salinity, nitrate, nitrite, ammonium, and phosphate	Coastal region Dongchong Bay	Fungi regulate phytoplankton bloom	[59]
Temperature, depth, salinity, riverine input, location	Upwelling ecosystem off the coast of Central Chile	Organic matter decomposition	[57]
Dissolved nitrogen, particulate phosphorous silicate, pH, salinity, chlorophyll *a*	Coastal water column	Spatial variations	[18]
Dissolved oxygen and depth	East China Sea water and sediments	Ocean currents govern assemblages	[54]
Temperature, pH, insolation, dissolved inorganic carbon	Waters of Piver’s Island Coastal Observatory (PICO)	Temporal variations	[21]
Depth, temperature, and dissolved oxygen	Epi- to abyssopelagic zones of the Western Pacific Ocean	Distinct zonation of assemblages in the water column	[23]
Salinity	Baltic Sea	Salinity threshold separates assemblages	[76]

**Table 4:** Abundance of planktonic fungi in various oceanic regions estimated by different methods and their comparison with bacterial abundance.

Estimation Method for Fungi	Sampling Region	Fungal Abundance	Bacterial Abundance	Reference
Biomass carbon	Coastal Chile	0.03–6 μg C L^−1^	-	[38]
Biomass carbon	Coastal Chile	0.01–40 μg C L^−1^	10–44 μg C L^−1^	[82]
Fatty Acid (18:2ω6)	Coastal Chile	0.1–3 μg L^−1^	10–44 μg C L^−1^	[82]
Ergosterol	Arctic waters	1.02 μg C L^−1^	5 to >25 μg C L^−1^	[24,83]
qPCR (DNA concentration)	West Pacific Warm Pool	Basidiomycota (max. 10 ng μL^−1^, open-ocean station) Ascomycota (max. 14 pg μL^−1^, coastal station)	~10 ng μL^−1^	[64]
qPCR (18S rRNA gene copy number)	Coastal Plymouth, Western English Channel	5.1 × 10^5^ to 9.9 × 10^7^ copies L^−1^	0.2 × 10^6^–1.6 × 10^6^ cells mL^−1^	[19,84]
qPCR (18S rRNA gene copy number)	Coastal region, Bohai Sea	4.28 × 10^6^ to 1.13 × 10^7^ copies L^−1^	~ 2 × 10^6^ cells L^−1^	[18]
qPCR (18S rRNA gene copy number)	PICO	1.0 × 10^7^ to 7.54 × 10^8^ copies L^−1^	-	[21]

“-” = data not available.

## Data Availability

Not applicable.

## Supplementary Materials

The following supporting information can be downloaded at: https://www.mdpi.com/article/10.3390/jof8050491/s1, Figure S1: Maximum-likelihood (ML) tree of the culturable fungi isolated from marine sediment. A total of 348 ITS sequences of culturable fungi isolated from sediments across the globe were retrieved from the NCBI Nucleotide database. Sequences in the tree were aligned with MUSCLE using default settings. Phylogenetic analysis was performed using FastTree2.1 software for the construction of the ML tree, which uses the Shimodaira–Hasegawa test to estimate the reliability of each split in the tree; Table S1: Culturable and molecular studies on fungi in the sediments of different oceanic regions. References [10,11,13,51,78] are cited in the supplementary materials.
