# Supplementary material for: Diversity, Abundance, and Ecological Roles of Planktonic Fungi in Marine Environments
Source: J Fungi (Basel). 2022 May 8;8(5):491. doi: 10.3390/jof8050491 (PMC9147564; doi:10.3390/jof8050491)
Supplement: Supplementary file 1 [file jof-08-00491-s001.zip › jof-1708869-supplementary.pdf]

Table S1. Culturable and molecular studies on fungi in the sediments of different oceanic regions.

Oceanic region	Type of sample	Reference
Bay of Bengal	Sediments	Velmurugan et al. (2013)
Arabian Sea	Oxygen minimum zone, sediments	Jebaraj et al. (2010)
Mariana Trench	Deep-sea mud	Takami et al. (1997)
Chagos Trench	Deep-sea sediments	Raghukumar et al. (2004)
Central India Basin	Deep-sea sediments (5000 m)	Damare et al. (2006); Singh et al. (2010); Singh et al. (2012)
East Indian Ocean	Deep-sea sediment (4000 m)	Zhang et al. (2014)
South China Sea	Deep-sea sediment (2400-4000 m)	Zhang et al. (2013)
Suruga Bay	Marine sub-surface sediments (3-40 mbsf)	Nagano et al. (2016)
White Sea	Bottom soil (10-30 m)	Khusnullina et al. (2018)
Delaware Bay	Sediment (2-4 cmbsf)	Burgaud et al. (2013)
Admiralty Bay	Sediment	Wentzel et al. (2019)
Canterbury basin	Deep-Subseafloor sediment	Rédou et al. (2015)
East Pacific Ocean		Wei et al. (2018)

[10,11,13,51,78]

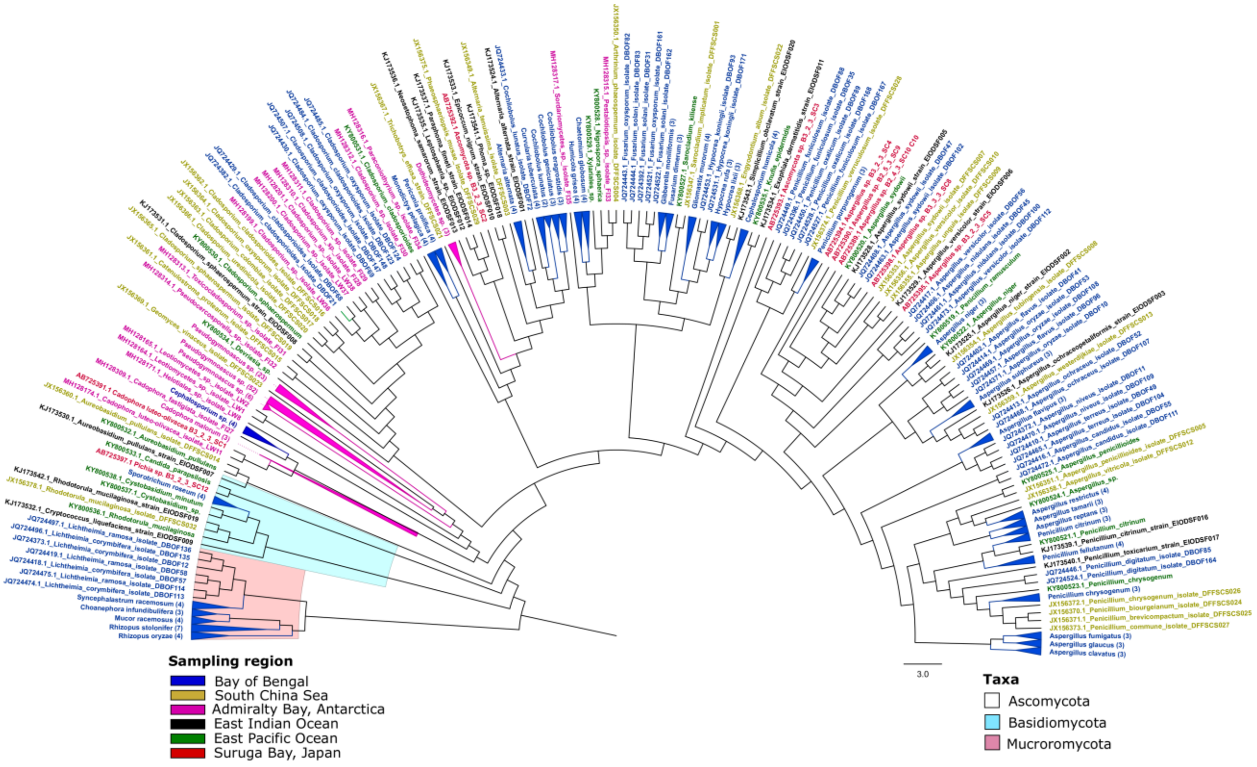

Figure S1. Maximum-Likelihood (ML) tree of the culturable fungi isolated from marine sediment. A total of 348 ITS sequences of culturable fungi isolated from sediments across the globe were retrieved from the NCBI Nucleotide database. Sequences in the tree were aligned with MUSCLE using default settings. Phylogenetic analysis was performed using FastTree2.1 software for the construction of the ML tree, which uses the Shimodaira-Hasegawa test to estimate the reliability of each split in the tree.
